# Correspondence

**Published:** 1892-03

**Authors:** T. M. McIntosh

**Affiliations:** Thomasville, Ga.


THOMASVILLE, GA., February, 1892.

DR. D. H. HOWELL:

In the journals which you sent me a few days ago containing my letters from Paris and Berlin in October, on page 492: "Pion, the originator of the Heamostacie in operation," should be Pean, the originatoı of the *Hœmostatic forceps* in operations.

On page 493, last line and first word of page 494, in speaking of the endoctreline of arteries in relation to formation of thrombus, it reads, "or disease of this *sort*," but should be or disease of this *part*.

*Mr. Armondale* is "Armondoli," and *Pean* is Pion.

In December number, page 596, *steam* sterilizer is "straw sterilizer." Page 598, in speaking of the methods of removing remaining lens substance in cataract operations, it reads, "and by upward pressure of the lid to reverse it," should be to *re move*, not "reverse."

There are a few others of spelling, which do not alter the sense, and due to my bad writing, that does not matter.

T. M. MCINTOSH.

